# Exploring the Modulatory Effects of Gut Microbiota in Anti-Cancer Therapy

**DOI:** 10.3389/fonc.2021.644454

**Published:** 2021-04-13

**Authors:** Wenyu Li, Xiaorong Deng, Tingtao Chen

**Affiliations:** ^1^ Department of Gastrointestinal Surgery, The Second Affiliated Hospital of Nanchang University, Nanchang, China; ^2^ Queen Mary School, Nanchang University, Nanchang, China; ^3^ National Engineering Research Center for Bioengineering Drugs and the Technologies, Institute of Translational Medicine, The First Affiliated Hospital, Nanchang University, Nanchang, China

**Keywords:** gut microbiota, neoplasms, cancer therapy, probiotics, fecal microbiota transplantation, diet therapy

## Abstract

In the recent decade, gut microbiota has received growing interest due to its role in human health and disease. On the one hand, by utilizing the signaling pathways of the host and interacting with the immune system, the gut microbiota is able to maintain the homeostasis in human body. This important role is mainly modulated by the composition of microbiota, as a normal microbiota composition is responsible for maintaining the homeostasis of human body, while an altered microbiota profile could contribute to several pathogenic conditions and may further lead to oncogenesis and tumor progression. Moreover, recent insights have especially focused on the important role of gut microbiota in current anticancer therapies, including chemotherapy, radiotherapy, immunotherapy and surgery. Research findings have indicated a bidirectional interplay between gut microbiota and these therapeutic methods, in which the implementation of different therapeutic methods could lead to different alterations in gut microbiota, and the presence of gut microbiota could in turn contribute to different therapeutic responses. As a result, manipulating the gut microbiota to reduce the therapy-induced toxicity may provide an adjuvant therapy to achieve a better therapeutic outcome. Given the complex role of gut microbiota in cancer treatment, this review summarizes the interactions between gut microbiota and anticancer therapies, and demonstrates the current strategies for reshaping gut microbiota community, aiming to provide possibilities for finding an alternative approach to lower the damage and improve the efficacy of cancer therapy.

## Introduction

Cancer is one of the leading causes of death worldwide. It arises as a result of accumulated genetic disorders that leads to dysregulations in cell cycle, having the potential to undergo unlimited times of division and imposing a strong negative impact on the normal physiological functions of the host (1). As the mutations accumulate, the life quality of the patient is largely impaired, and most importantly the life span is reduced (2).

Finding a cure for cancer to prolong the lifespan has long been the greatest challenge during the development of medical research. Over the years, researchers have worked out a variety of therapeutic methods against cancer, including chemotherapy, radiotherapy, immunotherapy, surgery and so on. Although these methods are able to inhibit the progression or even eliminate some types of cancer cells, there are still limitations due to acquired resistance as well as undesirable side effects caused by the low selectivity of anti-cancer agents between normal cells and cancer cells (3). For example, previous research findings suggest that collateral damage in the abdominopelvic region caused by radiotherapy can lead to bowel injury (4), and 80% of cancer patient suffer from chemotherapy-induced gastrointestinal toxicity (CIGT), with symptoms of diarrhea and abdominal pain (5). As these adverse effects seriously interfere with the anticancer therapy, finding an adjuvant method to ultimately overcome these complications becomes urgent for the clinical research.

The human microbiota consists of microoganisms (bacteria, archaea, fungi and viruses) present in the epithelial barriers of the host (6), with the most abundant being the commensal bacteria that coexist with human cells in the gastrointestinal tract (7). With the development of high-throughput sequencing technology, the composition of gut microbiota can be clearly identified. It mainly consists of 5 bacteria in healthy individuals: *Firmicutes*, *Bacteriodes*, *Actinobacteria*, *Proteobacteria* and *Fusobacteria* (8). This microbiota profile stays relatively unchanged throughout life once established (9), forming a unique “signature” in each individual with important functions associated with both innate and adaptive immune systems (9). In recent years, the gut microbiota has increasingly come under focus due to its impact on many human diseases, including diabetes, obesity, psychiatric disorders and gastrointestinal diseases (10). They regulate the balance between health and disease by maintaining local homeostasis to systematically regulating metabolism, hematopoiesis, inflammation, immunity and preventing pathogen infection (9), and the host can in turn “communicate” with the microbiota with the aid of several host molecules such as host microRNA (miRNA), hormones, cytokines, metabolites and metabolic signaling pathways (11). Additionally, recent insights importantly highlighted the impact of gut microbiota on responses across several cancer therapies (12), suggesting that regulating gut microbiota may improve the effectiveness of many cancer treatments, with a reduced cytotoxic activity.

This article reviews how the gut microbiota, as an adjuvant therapy, affect the efficacy of four anti-cancer therapies including chemotherapy, radiotherapy, immunotherapy and surgery, at the same time reduce the adverse effects. These manipulations may be conducive to the promotion of personalized medicine and effective anti-cancer treatment.

## The Impact of Gut Microbiota on Oncogenesis and Tumor Progression

There are many factors contributing to the oncogenesis of cancer, and the study of these oncogenic pathways have clearly given us insight into the nature of this devastating disease. In 2000, Hanahan and Weinberg presented six hallmarks of cancer, and updated in 2011 with two emerging hallmarks (13). By acquiring these properties, normal cells can undergo tumorigenic transformation and, in the end, become cancer cells. The hallmarks are: i) self-signaling for proliferation, ii) evading anti-growth signals, iii) invasion and metastasis, iv) immortality, v) angiogenesis, vi) resisting apoptosis, vii) deregulating energy metabolites, viii) evading immune system. The main mechanism by which normal cells gaining these cancer hallmarks are accumulated mutations in the genome, including somatic structural variants (SVs) and copy number alterations (CNAs), that interfere with the normal regulatory controls (14). In recent years, increasing researches have revealed the relation between microbiota (especially the gut microbiota) and carcinogenesis, suggesting that the gut microbiota can be involved as an environmental factor and contribute to genetic alterations as well.

The indirect bacterial mechanism of oncogenesis is represented in the process of chronic inflammations induced by bacterial infection. In this case, the microbiota chronically generates several inflammation mediators such as Tumor Necrosis Factor-α (TNF- α) and Interleukin-1 (IL-1), which further lead to the induction of transcription factor nuclear factor-κB (NF-κB) and contribute to carcinogenesis (15). In addition, the bacteria-induced oncogenesis could also be direct through the effect of microbial metabolites or toxins. Previous studies have shown that several strains of gut microbiota are responsible for the tumorigenesis of different cancer types, such as gastric cancer, colorectal cancer (CRC) and hepatocellular carcinoma (15, 16). Their carcinogenic processes are all linked to the production of microbial metabolites. the carcinogenic process of gastric cancer, CagA proteins produced by H. pylori are transferred into gastric epithelial cells and interact with pro-oncogenic phosphatase SHP2 and the polarity-regulating kinase PAR1/MARK, driving the host signaling pathways that favors carcinogenesis (17). *Bacteroides fragilis* is a strong risk factor of CRC, which could act as an opportunistic pathogen (15). In antigen-presenting cell (APC) mutant mice model which are predisposed to intestinal cancer formation, the enterotoxigenic *B. fragilis* (ETBF), one of the two subtype of *B. fragilis*, can induce colitis and inflammatory bowel disease (IBD) through the pathway of β-catenin/Wnt/NF-κB signaling, and further lead to the oncogenesis of CRC (15). On the other hand, *B. fragilis* toxin (Bft) can up-regulate spermine oxidase (SMO) in colon epithelial cells, causing reactive oxygen species (ROS) production and indirect DNA damage (18, 19). Other microbial metabolites associated with carcinogenesis include *Pasteurella multocida* toxin, cytolethal distending Toxin (CDT) (15) and inositol phosphate phosphatase D (IpgD) (16). These could all contribute to the cell transformation, in which the normal cell responses are altered, and further elevate the risk for developing cancer.

Given the association between the gut microbiota and carcer development, it should be considered that a healthy gut microbiota profile is both sufficient and necessary for maintaining a healthy microenvironment. Therefore, by targeting dysbiosis, the efficacy of some anti-cancer therapies may be improved, with a better prognosis and reduced side effects.

## The Interplay Between Gut Microbiota and Cancer Therapy

### Microbiota and Chemotherapy

Chemotherapy is one of the most potent approach to treat cancer systematically at present. As the chemotherapy drugs can be delivered through blood circulating system, it can act on hematopoietic malignancies or tumor with metastasis (20), targeting DNA, topoisomerase or tubulin to prevent the growth and proliferation of cancer cells (21). However, due to the lack of specific targets of chemotherapy drugs, there are still unavoidable complications caused by cytotoxic effect. In further studies, the mechanisms of chemotherapy toxicity revealed a bidirectional interaction between gut microbiota and cytotoxic drugs.

#### The Influence of Chemotherapy on the Gut Microbiota: Composition and Translocation

The chemotherapy-induced change in microbiota composition has been widely studied in a considerable number of pre-clinical models, demonstrating a decreased total number and diversity. Although different chemotherapy drugs may exert different influences (22, 23), the overall impact was concluded as a reduced *Lactobacillus* and *Bifidobacterium*, together with an increased *Escherichia coli* (E. coli) and *Staphylococcus*, consisting with the result of clinical studies (5). This disruption in microbiota composition is associated with an activated inflammatory pathway and an impaired barrier function, which makes the host more vulnerable to pathogens (5, 22).

In addition to changes of microbiota composition, chemotherapy can also induce microbiota translocations, which is often due to the injured epithelium of the gut (24). During this process, the gram-positive bacteria strains, such as *Lactobacillus johnsonii*, *Lactobacillus murinus* and *Enterococcus hirae*, are transferred by the circulation system to peripheral lymphoid organs such as mesenteric lymph nodes and spleen (25). There, the microbiota facilitates the stimulation of memory T helper 1 (Th1) and the conversion of naïve CD4^+^ T cells to T helper 17 (Th17) that secrete IL-17, together with an increased production of other secreting molecules such as interferon gamma (IFN-γ) which further contribute to the healing of mucosa and anticancer responses (25).

#### The Direct Influence of Microbiota on Chemotherapy: Drug Pharmacokinetics and ROS Production

**Figure 1:**
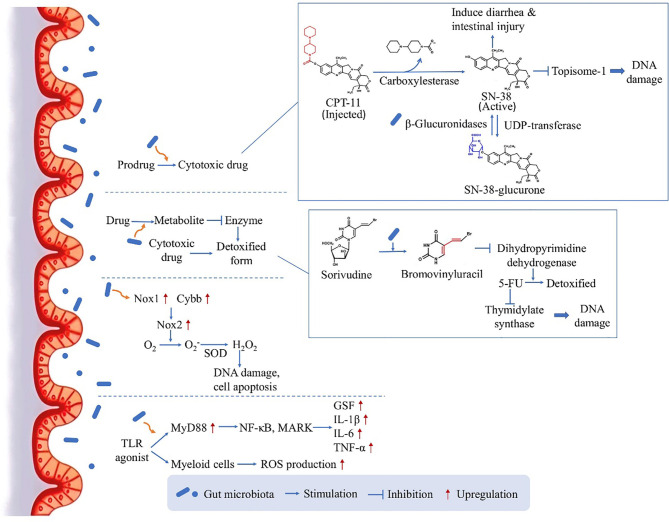
The direct influence of microbiota on chemotherapy: drug pharmacokinetics and ROS production. By several mechanism pathways, the gut microbiota can directly act on the drug conversion and gene transcription, leading to an either enhanced therapeutic effect or enhanced side effect.

As the influence between gut microbiota and chemotherapy is bidirectional, the microbiota can in turn affect the efficacy of chemotherapy (**Figure 1**). One of the mechanisms is that, orally administrated drugs and some injected drugs depend on gut microbiota to be converted into active form to exert the anticancer function (6, 26). For example, CPT-11 (Irinotecan) is a prodrug administrated intravenously in CRC treatment, and is converted into its active form SN-38 by carboxylesterase (26). The active drug works as topoisome-1 inhibitor, which induces single and double strand breaks of the DNA by blocking DNA ligation, leading to tumor cell death (26). Then the drug is detoxified by uridine diphosphate-glucuronosyl transferase (UDP-transferase) (26). However, experiment showed that the amount of SN-38 was elevated from 2% of administered dose to 12% in feces (27), because the intestinal microbiota-produced β-glucuronidases are able to convert the detoxicated SN-38-glucurone back to its active form SN-38 by deconjugation, and the increased concentration of SN-38 in the colon could cause diarrhea and intestinal injury (28). The result verified the role of microbiota in drug pharmacokinetics, providing a potential target for reducing side effects.

On the other hand, gut microbiota can facilitate the production of drug metabolite which inhibits a critical enzyme used for the detoxification of another drug, leading to enhanced side effects. Many studies have focused on the toxicity of 5-fluorouracil (5-FU) induced by microbiota, for example if Sorivudine and 5-FU are taken together by rats, Sorivudine would be metabolized to bromovinyluracil, and further inhibit the enzyme dihydropyrimidine dehydrogenase responsible for the detoxification of 5-FU (26). As 5-FU is an inhibitor of thymidylate synthase critical in DNA replication, the increased duration and concentration of 5-FU in the body will cause serious systemic effects including diarrhea and even reduction in leukocytes and platelets (29–31). Interestingly, this conversion only happened *in vivo*, especially in the intestinal contents, and it was then confirmed by experiment that gut microbiota species was responsible for the production of bromovinyluracil (32), indicating the key role of gut microbiota in the chemotherapy-induced toxicity.

Moreover, in contrast to increasing chemotherapy-induced toxicity, the gut microbiota could also facilitate the anticancer activity of chemotherapy drugs. This is achieved through inducing the expression of enzymes which are responsible for ROS production. Oxaliplatin, a commonly used chemotherapeutic agents, can generate ROS in tumor cells to induce cell apoptosis by damaging DNA (33). According to experiment results, the anticancer effect will be reduced in germ free mice or if the mice are treated with antibiotic cocktail (ABX), as the impaired microbiota function could lead to reduced expression levels of Nox1 and Cybb genes coding for NADPH oxidase 2 (Nox2) (34). This altered gene expression would contribute to compromised therapeutic effect, as Nox2 can transfer electrons to generate superoxide $\left(O_{2}^{-}\right)$ and further lead to H_2_O_2_ production by an enzyme called compartment-specific superoxide dismutase (SOD), having the ability to induce DNA damage in tumor cells and permit cell apoptosis (33). Furthermore, there is also a second pathway by which gut microbiota could facilitate the myeloid cell-produced ROS through Toll-like receptor (TLR) agonist releasing and the downstream expression of myeloid differentiation primary response gene 88 (MyD88) (34). This pathway further activates NF-κB õand MAPKs and induces the expression of several genes coding for inflammatory cytokines, including granulocyte colony-stimulating factor, Interleukin-1β (IL-1β), Interleukin-6 (IL-6) and TNF-α (35). This study substantiated the ROS-generating pathway used by microbiota to modulate tumor microenvironment and further affect the outcome of chemotherapy, highlighting a mechanism for obtaining optimal anticancer responses.

#### The Indirect Influence of Microbiota on Chemotherapy: The Immune System

As the microbiota and chemotherapy drugs can both act on the immune system, they could use it as a medium to interact with each other. When the component of gut microbiota is changed by the chemotherapy as a side effect of the treatment, the alteration can further influence the function of innate immune system by reducing APCs (36) and produce inflammatory cytokines (37), which lead to the progression of Chemotherapy-induced gastrointestinal toxicity (CIGT) (5). Furthermore, certain phyla of microbiota could play an indispensable role in anticancer chemotherapy by regulating the immune response. One of the chemotherapeutic agents, Cyclophosphamide (CTX), is able to exert systemically anticancer effect through inducing naïve CD4^+^ T cells to Th1 and Th17 fate (25). This process is microbiota-dependent: the entry of symbiotic bacteria (such as *Lactobacillus johnsonii* and *Enterococcus hirae*) into mesenteric lymph nodes promotes the induction of Th17 and Th1 memory responses in the spleen in the presence of bone marrow-derived dendritic cells, performing tumor antigen cross-presentation with the help of TLR and its adaptor MyD88 (25). It is critical for achieving successful therapeutic outcome of CTX (25). Similarly, another experiment demonstrates that the colonization of *Barnesiella intestinihominis* in the colon is able to modulate tumor microenvironment, promoting Interferon-γ (IFN-γ) production and reducing Treg cells (38). In this research, the immunogenic microbiota is referred to as “oncomicrobiotics”, cooperating with CTX against many types of cancers by unknown mechanisms (38).

#### Gut Microbiota Induces Chemoresistance

Resistance and heterogeneous responses to chemotherapeutic drugs are a major challenge in cancer treatment (39). Currently, researchers have identified several different intrinsic cellular mechanisms involved in chemotherapeutic resistance, and the microbiota-induced drug resistance has gradually received attention in recent years.

Based upon the fact that in recurrent CRC patients the microbiota *Fusobacterium nucleatum* (Fn) is especially enriched (40), it is found that this microbiota phyla is able to induce chemoresistance in a FadA-dependent manner: the oncogenic and inflammatory pathway are stimulated by the binding of FadA and E-cadherin and the downstream β-catenin signaling, which further elevated the expression of transcription factors and genes including oncogenes and Wnt, resulting in increased inflammation and tumor cell growth (41). This study pointed to the potential role of Fn in chemoresistance, indicating that the colonization of specific phyla of microbiota could be a general characteristic of cancer patients.

In addition to utilizing the signaling pathways of the host, the gut microbiota could also induce chemoresistance by inactivating the chemotherapy drug. For example, *Gammaproteobacteria* is a microbiota species especially abundant in the duodenum, and could convert the chemotherapy drug gemcitabine (a chemotherapy drug used to treat cancers of pancreatic, lung, breast or bladder) to its inactive metabolite by expressing a long isoform of the enzyme cytidine deaminase (CDD_L_), contributing to the drug resistance.

Altogether, these research findings underline the importance of gut microbiota in the development of chemoresistance, which may provide an alternative target to deal with this obstacle.

### Microbiota and Immunotherapy

Immunotherapy is a promising therapeutic method for cancer treatment, acting on cancer types which develop resistance to conventional anti-cancer therapies (6). It targets cancer cells with the aid of host immune system, and has already proved to be effective in many clinical trials (42). The first attempt of cancer immunotherapy dates back to 1890s when Coley developed the first cancer vaccine, and the approval of the first immune checkpoint inhibitor in 2011 represents a new era of anti-cancer treatment (43). The recent decades witnessed a surge in exploring various methods for anti-cancer immunotherapy. Some of the promising immunotherapy methods include adoptive T cell transfer (i.e., transferring cytotoxic T cells which are tumor-specific to patients), CpG-oligodeoxynucleotide (i.e., a TLR9 agonist, containing unmethylated CG dinucleotide and has immune stimulation similar to bacterial DNA, which triggers the body’s defense mechanism and causes obvious and diversified immune response through a series of signal cascade transduction), immune checkpoint inhibitors (i.e., antibodies that target immune checkpoints to prevent tumor cells from escaping antitumor immunity, which has proved to be effective in advanced and metastatic cancer) (6). The US Food and Drug Administration has approved three immune checkpoint inhibitors: cytotoxic T lymphocyte-associated antigen-4 monoclonal antibody (CTLA-4), programmed cell death protein-1 (PD-1), and programmed cell death-ligand 1 (PD-L1) (44). Though they act *via* different mechanisms, the rationale is the same: to block the protective signaling pathway of Tregs hijacked by tumor cells, and reactivate the suppressed immune effectors. This can further restore the immune responses against cancerous cells when T cells are exhausted by the chronic activation of tumor antigen, achieving a better therapeutic outcome (45). As the gut process a great number of innate and adaptive immune cells, the interactions between immune cells and the commensal gut microbes could contribute to a robust immune response which protect the body from pathogens (46).

#### Gut Microbiota Influences the Immune System and Immunotherapy

In general, the impact of gut microbiota on the immune system can be involved in all anticancer therapies. The effects can be local, which is restricted to the gut mucosa, or can be systemic, which is due to primed dendritic cell that travel through circulation system (47). The local tolerance is mediated by the induction of Tregs *via* several signaling pathways, including interleukin 10 (IL-10), polysaccharide A and TLR (47). Short chain fatty acids (SCFAs) produced by microbiota are also able to affect local immunity *via* IgA, contributing to an enhanced immunity (48). In contrast to the local effect, the distant effects of gut microbiota on immunity requires another mechanism named “cancer-immunity cycle” model, which depends on the tumor antigen-activated T cells for recognizing and killing tumor cells (49).

There are also associations between specific strains of gut microbiota and the development of immune cells. *Segmented filamentous* bacterium (SFB) can induce CD4^+^ T helper cell fate as well as an increased resistance against *Citrobacter rodentium* (50), and *Clostridium* strain contributes to the differentiation of CD4^+^ T regulatory cells (51). Similarly, dendritic cells can also be regulated by gut microbiota, through the process of cytokine secretion, antigen presentation and T cell activation (52).

In addition to the impact on host’s original immune responses, it is also suggested that the composition of intestinal microbiota may influence the response of several immunotherapies such as immune checkpoint inhibitors (52, 53). Previous studies have indicated that the anti-CTLA activity is related to *Bacteroides*, while the effect of anti-PD-L1 is *Bifidobacterium*-dependent (54). The anti-CTLA therapy could not exert its function in germ-free mice or mice treated with antibiotics, but this situation could be improved by orally feeding the mice with *Bacteroides thetaiotaomicron*, *Bacterooides fragilis* or *Burkholderia cepacia* to induce dendritic cell and IL-12-dependent Th1 cell responses. One of the mechanisms that contribute to restoration of anti-CTLA activity is that *Bf* could utilize the TLR2/TLR4 signaling pathways to activate immunoprotection. The distribution of *Bf* on the intestinal mucosa is also responsible for the microbiota-dependent immunomodulatory effects of CTLA-4 antibody (53). In 2019, 11 strains of bacteria isolated in the gut of human were shown to possess the ability to facilitate immune checkpoint inhibitors by inducing IFN-γ^+^ CD8^+^ T cells, which strains include *Parabacteroides* spp., *Alistipes senegalensis*, five *Bacteroides* spp., *Eubacterium limosum*, *Ruminococcaceae bacterium cv2*, *Phascolarctobacterium faecium* and *Fusobacterium ulcerans* (55). Collectively, these research findings suggest that the immunostimulatory function of immunotherapy is strongly microbiota dependent.

#### The Immunotherapy Affects Gut Microbiota

From another point of view, the immunotherapy can in turn alter the composition of gut microbiota. The anti-CTLA-4 treatment is able to induce decrease in *Bacteroidales* and *Burkholderiales* and increase in *Clostridiales*, whereas the amount of *Bacteroides fragilis* is relatively unchanged (6). Similarly, in a study using anti-PD-1 to treat patients with melanoma, the abundant of different strains of gut microbiota was altered after the therapy, with an increase in *Clostridiales/Ruminococcaceae* in responders and an increase in *Bacteroidales* in non-responders (56). Other immunotherapy methods like allogeneic stem cell transplant (allo-HSCT) can also alter the abundance of *Enterococcus*, *Streptococcus* and *Proteobacteria* (57). As a result, the altered composition of gut microbiota caused by the exposure to immunotherapy can induce a negative impact on the effectiveness of further treatment, including colitis, thyroid dysfunction and even autoimmune disease in which the enteric bacteria become the target of host antibodies (53).

### Microbiota and Surgery

For solid cancers, especially in the situations which the tumor is in the early stage or with no metastasis, performing surgery to remove the neoplasm lesion can be an effective treatment (58). In these cases, the potential influence of intestinal microbiota on surgical outcomes is possibly due to the direct interaction between intestinal microbiota and the site of resection. This association has been demonstrated mostly in CRC. Although the therapy for CRC is usually multidisciplinary, the major surgical treatment for non-metastatic or locally advanced rectal cancer is total mesorectal excision (TME) (59). The research findings have shown that after surgery, the amount of some obligate anaerobes which are responsible for gastrointestinal homeostasis such as *Clostridium coccoides*, *C. leptum*, *B. fragilis*, *Bifidobacterium*, *Atopobium* and *Prevotella* are reduced, together with an increase in pathogens including the *Facultative anaerobes*, *Enterobacteriaceae*, *Enterococcus*, *Staphylococcus* and *Pseudomonas* (60). In turn, this disturbed gut microbiota community could affect the outcome of the therapy, responsible for an increased recurrence rate and a decreased disease-free survival (60).

In addition to dysbiosis, anastomotic leak (AL) is also a most common life-threatening complication after CRC. Despite improvements in perioperative medical care, the AL rate has remained between 1% and 19% over the past few decades (61, 62) while little is known about the microbial characteristics and mechanisms associated with AL. Then in 2013, Stern et al. first demonstrated that harmful intestinal factors (such as bacteria) may invade the intestinal tissue when the epithelial barrier is impaired, which may delay the healing of the anastomosis and lead to AL (63). This suggests that anastomotic healing after colorectal surgery is greatly depend on the restoration of epithelial barrier integrity. Recently, van Praagh et al. employed 16S MiSeq sequencing on colorectal anastomosis tissue samples and reported that AL development was associated with a low microbial diversity, which was characterized by a high abundance of the dominant Lachnospiraceae and Bacteroidaceae families and a low abundance of *Prevotella oralis* (64). Collectively, these experimental phenomena shed light on the importance of gut microbiota manipulation during post-operative care, suggesting that it is the part that should not be ignored.

### Microbiota and Radiotherapy

Radiotherapy is a commonly used anticancer therapy using ionizing irritation to generate reactive chemical species such as ROS or reactive nitrogen (RNS). It directly induces DNA damage, including single-strand breaks and double-strand breaks, through energy transfer (6, 65–67). Radiotherapy is also commonly associated with immunogenic process, as it can induce immunogenic cell death (ICD) which evokes subsequent immune responses by antigen presenting cells and cytotoxic T cells. The close association of radiotherapy and immunotherapy allows radiotherapy to not only deal with local lesions, but also have a systemic effect for treating distant malignancies *via* migrating DC and cytotoxic T cells, known as abscopal effect. Therefore, radiotherapy and immunotherapy are often combined to achieve a better efficacy (67).

The side effects induced by total body irritation (TBI) mainly result from the fact that radiotherapy could affect tumor cells and surrounding normal cells alike. Inflammation is a common consequence of radiation irritation not only due to weakened immune system, but also caused by altered gut microbiota. The reduced integrity of gut epithelium leads to microbiota translocation to mesenteric lymph nodes, together with an increased lipopolysaccharide (LPS) derived by microbiota (68). Similarly, the radiotherapy used for treating head and neck cancer such as nasopharyngeal carcinoma can lead to oral mucositis, compromising the anticancer therapy (69). On the other hand, several studies have also proved that radiation irritation is able to induce reduction in microbiota diversity (70–72). In a pilot study of three pediatric cancer patients with pelvic rhabdomyosarcoma, the radiotherapy and antibiotic treatment caused a decreased abundant of *Firmicutes* and increased *Proteobacteria* (73).Compared to the healthy controls and the patients who didn’t undergo microbiota change after radiotherapy, the patients with dysbiosis are prone to develop pathologic conditions like diarrhea (72), inflammatory bowel disease (IBD) and type 2 diabetes (T2D) (70). Moreover, the gut microbiota could also affect the therapeutic effects of radiotherapy. A preclinical study revealed the presence of gut microbiota is responsible for increased radio-sensitivity of the intestinal endothelium, showing that the production of angiopoietin type 4 in germ free mice model could lead to reduced endothelial cell apoptosis and lymphocyte infiltration (74, 75). Further explorations are promising for discovering the mechanisms and potential therapeutic modulation of gut microbiota on the therapeutic effects of ionizing radiation.

## Manipulating Gut Microbiota to Achieve Better Therapeutic Efficacy

There are lines of evidence implicating that different patients respond differently to anticancer therapy. The reasons are concluded as different host genes, different tumor mutations, different environmental factors, and to some extent the different gut microbiota composition (52). Therefore, the manipulation of gut microbiota could be an effective method to improve the efficacy of conventional anticancer therapy.

### Probiotics

According to the research findings discussed in previous sections, those mechanism pathways could be considered in combination therapy for cancer treatment. On the one hand, reducing the amount of genus that could impair the efficacy of anticancer therapy, for example providing antibiotics to target the strains that could induce resistance, is a goal of microbiota-targeted combination therapy. On the other hand, as dysbiosis is one of the side effects brought by anticancer therapy, targeting the abnormal microbiota profile by providing patients with beneficial bacterial strains can also effectively reestablish the microbiota community, and further restore the abilities of microbiota involved in drug-microbiota interaction (76, 77).

Given these notions, the use of probiotics has become a significant research field. Probiotics refer to the live bacterial species introduced into human body, exerting their beneficial effects by reestablishing the normal microbiota community (78). Much attention has been paid to the effect of probiotics on tumor-treatment-related toxicity (79) and its potential in improving the efficiency of cancer treatment (80). According to research findings, the preoperative administration of probiotics, prebiotics and synbiotics (the combination of probiotics and prebiotics) effectively attenuates the post-operational infection, with a reduced inflammation, morbidity and hospital stay (81, 82). This is achieved by modulating the composition of microbiota and improve the intestinal barrier (82). Additionally, the use of probiotic nutrition strategies has also been proved to be effective against radiotherapy-induced side effects through enhancing immune response, including the administration of probiotic Bifico against chemoradiotherapy-induced oral mucositis (69) and the use of “designer probiotics” in CRC and breast cancer (47). Notably, there is emerging evidence suggesting that the administration of specific bacteria strains, such as *Lactobacillus* spp. and *Bifidobacteriales*, is associated with better anticancer efficacy. A recent clinical trial showed that providing probiotics containing *Lactobacillus* and *Bifidobacteria* to post-operative CRC patients for six mouths effectively reduced the expression of many pro-inflammatory cytokines, including TNF-α, IL-6, IL-10 and IL-12, but the level of IFN-γ is relatively unchanged (83). In mice with adverse intestinal microbiota, oral probiotics containing *Bifidobacterium* could restore the anti-tumor effect of PD-L1 blockade, mainly by promoting the maturation of dendritic cells so as to improve the activity of tumor-specific CD8^+^ T cells (52). Similarly, the decreased Firmicutes/Baxteroides ratio also leads to a decreased tumorigenic outcome (84).

Looking into the mechanisms, it is found that *Bifidobacteria* could act on the host immune system through the IFN-γ pathway (52). By treating the mice with probiotic Bifidobacteria, the number of major histocompatibility complex-II (MHC-II) dendritic cells within the tumor were elevated due to the secreted costimulatory molecules (85), together with the tumor specific T cells, both in periphery and in tumor. On the other hand, *Bifidobacterium* spp. can activate the transcription of up to 760 genes in tumor-infiltrating dendritic cells which are related to antitumor responses, such as Cd70 and Icam1 gene for CD8^+^ T cell activation, Relb for dendritic cell maturation, and Rab27a for antigen processing and cross presentation (52).

In addition to the direct interaction of bacteria and immune system, the probiotics can also exert its function by secreting several probiotic-derived molecules. The effector molecules that have been shown to be associated with anticancer property are competence and sporulation factor (CSF), inorganic polyphosphates, ferrichrome, and some other peptides such as P75 and P40 (86). These secreting molecules can act through different mechanisms and pathways. CSF is a type of quorum-sensing pentapeptide, and is able to induce the upregulation of heat shock proteins (Hsps). This further activates epithelial cell survival pathway of protein kinase B/Akt and p38 MAP kinase by organic cation transporter 2 (OCTN2). The inorganic polyphosphate can also induce Hsps expression and act on the integrin β_1_-p38 MAPK pathway, and the peptides P75 and P40 is associated with the activation of Akt cell survival pathway. The molecule ferrichrome, derived from probiotic *Lactobacillus casei*, can selectively act on colon cancer cells to induce the cleaving of Caspase-3 and PARP and activate the apoptosis pathway through DDIT3-JNK signaling-mediated ER stress response pathway, with a therapeutic effect even better than cisplatin and 5-FU (86).

### Fecal Microbiota Transplantation (FMT)

The concept of fecal microbiota transplantation (FMT) was initially established for treating *Clostridium difficile* infection (CDI). In 1958, Eiseman and colleagues first described this therapeutic method for presumed severe CDI in a case series, which is to transplant functional microbiota from healthy individuals into the gastrointestinal tract of patients to rebuild the normally functioning intestinal microbiota (87, 88). It was not until 2012 that FMT was first linked to cancer treatment by Neemann et al., and in this case by performing FMT, a patient with acute lymphocytic leukemia (ALL) successfully recovered from severe CDI induced by the immunocompromised condition after allogeneic hematopoietic stem cell transplant (89). Later, this therapy was put into practice in the treatment of many other hematological malignancies, in which immunocompromised condition and dysbiosis often occurred as a post-transplantation complication, leading to *C. difficile* overgrowth and symptoms like diarrhea, abdominal pain and hematochezia (90, 91). Clinical trials of the use of FMT in the treatment of cancer patients are still in their early stages, but has proved its effect on many types of complications during anticancer treatment, including CDI that are resistant to traditional therapies (92), graft-versus-host disease after allogeneic stem cell transplantation (93), inflammatory bowel disease (94) and active ulcerative colitis (95). However, in some cases post‐FMT complications such as bacteremia may occur (91), and the mechanism is still unclear. Further studies are required to identify the risk factors for FMT and improve the safety.

### Dietary Factors

#### Short Chain Fatty Acids (SCFA)

Dietary factors have been considered to play a vital role in human health and disease for centuries. Over the last decade, there have been increasing interests in the research on the interplay between diet and the gut microbiota, and it is now widely accepted that gut microbiota can be shaped by dietary factors, leading to enriched beneficial microbiota strains and the production of SCFA. Generally, SCFA has an anti-inflammatory and anti-tumorigenic effect, but there are also exceptions in which specific SCFA could induce different outcomes. For example, being the focus of many studies, the butyrate, one of the SCFA, has a tumor suppressing effect (96), while acetate is a metabolite that has shown to be potentially oncogenic.

Being one of the dietary factors, dietary fibers have shown to effectively prevent CRC with an anti-inflammatory property due to its effect of maintaining the amount of microbiota that produce butyrate (47, 97). Another dietary factor is resistant starch, also a contributing factor of decreased risk of CRC and colitis. It can be converted to SCFA by fermentation in the large intestine, and can lead to reduction in gene expression associated with immune responses and inflammatory conditions including cyclooxygenase 2 (COX-2), NF-κB, IL-1β and TNF-α, having an anti-tumorigenic effect by either activating the expression of G-protein coupled receptor 43 (GPR43) which induces anti-inflammatory property, or inhibiting histone deacetylase (97). In addition, it can inhibit the cell proliferation by inhibiting the translocation of β-catenin from the membrane into the nucleus, preventing the downstream expression of growth factors related to cell growth (97). These findings have strong implications in searching an alternative approach to shape gut microbiota with dietary factors, which can be easily controlled by patients even in everyday life.

#### Vitamin D

Previous studies have demonstrated that vitamin D has an important immunomodulatory function. Several immune cells (such as T cells, B cells, neutrophils and APCs) express vitamin D receptor, allowing vitamin D to regulate the balance between pro-inflammatory and anti-inflammatory state (98). It can also mediate the antimicrobial peptide (CAMP) expression downstream of TLR activation, leading to phagosome formation and antimicrobial activity against pathogens (98).

In addition, vitamin D has shown to be effective against complications caused by radiotherapy *via* restoring the population of gut microbiota and reducing the number of opportunistic pathogens (99). In induced colitis, the vitamin D deficient mice have the characteristics of a reduced antimicrobial activity of angiogenin-4 protein (Ang4) (100).The relationships among vitamin D, gut microbiota and radiation-induced resistance were described as a “love-hate triangle”, indicating that these three factors could interact with each other during the process of the anticancer therapy (99). However, further studies are still needed for the understanding of the molecular mechanisms.

## Conclusions and Future Directions

As cancer is the leading cause of death worldwide, finding a cure for this devastating disease has long been a challenge for the research field. The current anticancer therapies mentioned above have proved to be effective in providing curative or palliative managements against cancer, but there are still several side effects during this process, leading to reduced efficacy and prognosis. Reports on the role of microbiota in cancer, combined with preclinical and clinical research, have led to the revelation of this topic as a potentially dominant mediator in response to cancer treatment. With the rapid development of the understanding of human gastrointestinal microbiota, there exists a close symbiotic relationship between the gastrointestinal microbiota and the host. In the context of many diseases of the digestive system, the disturbance of the composition of the gastrointestinal microbiota can be observed. Whether gastrointestinal microbiota imbalance is the cause or outcome of the disease, it may exacerbate the disease progression and influence the associated treatment strategies. In addition, it is demonstrated that the cancer treatment response can be enhanced by modulating the intestinal microbiome such as providing beneficial bacteria strains as probiotics or preforming FMT, and future therapy could utilize these methods to achieve a precise regulation of the microbiota composition, such as the specific amount of a particular microbiota genus. However, it is not clear which intestinal microbiome composition is best suited to promote the anti-tumor immune response, which needs to be carefully tested through clinical trials. It is also necessary to find out other important factors to modulate the intestinal microbiome, such as adjustment of preparation before the use of antibiotics. Only by fully understanding these mechanisms can we better optimize the regulation of the intestinal microbiota, and improve the potential for immune surveillance and cancer treatment.

## Author Contributions

TC conceived the idea for the review and designed its framework. WL and XD conducted the research and wrote the manuscript. All authors edited the manuscript. All authors contributed to the article and approved the submitted version.

## Funding

This study was supported by the National Natural Science Foundation of China (Grant no. 82060638, 81960103), Academic and technical leaders of major disciplines in Jiangxi Province (Grant no. 20194BCJ22032), and Double thousand plan of Jiangxi Province (high end Talents Project of scientific and technological innovation).

## Conflict of Interest

The authors declare that the research was conducted in the absence of any commercial or financial relationships that could be construed as a potential conflict of interest.
